# ARACOV-02. Specialized nutritional intervention and telerehabilitation in patients with long COVID: Protocol of a randomized controlled trial

**DOI:** 10.1371/journal.pone.0321811

**Published:** 2025-04-29

**Authors:** Beatriz Carpallo-Porcar, Carolina Jiménez-Sánchez, Sandra Calvo, Pilar Irún, Elena Kolesnyk-Sumskaya, Ana Isabel Aller-Blanco, Esther del Corral Beamonte

**Affiliations:** 1 Department of Physical Therapy, Faculty of Health Sciences, Universidad San Jorge, Zaragoza, Spain; 2 IIS Aragón, Zaragoza, Spain; 3 Department of Physiatry and Nursing, Faculty of Health Sciences, University of Zaragoza, Zaragoza, Spain; 4 Centro de Investigación Biomédica en Red en Enfermedades Hepáticas y Digestivas (CIBEREHD), Madrid, Spain; 5 Centro de Salud Pirineos, Huesca, Spain; 6 Centro de Salud Ensanche, Teruel, Spain; 7 Hospital Royo Villanova, Zaragoza, Spain; LAU Gilbert and Rose-Mary Chagoury School of Medicine: Lebanese American University School of Medicine, LEBANON

## Abstract

**Background:**

It is estimated that at least 10% of the population infected with SARS-CoV-2 develop Post COVID Condition, which is characterized by a diverse array of symptoms including dyspnea, fatigue, anxiety, depression, and deterioration in quality of life. The SARS-CoV-2 virus can trigger an excessive immune response, characterized by the release of pro-inflammatory cytokines including IL-6, IL-1, TNFα and reactive oxygen species. Specialized Pro Resolving Mediators (SPMs) (17-HAD, 14-HAD and 18_HEPE) that could be useful in Post COVID Condition modulating the inflammatory response. The objective is to determine the change in quality of life, inflammatory profile, functional capacity and emotional variables in a group taking a nutritional supplement (SPMs) plus a telerehabilitation programme.

**Methods:**

ARACOV-2 study is a double-blind, parallel-group, randomized control trial with two parallel interventions: Nutritional supplement and telerehabilitation vs placebo supplement and telerehabilitation. The primary endpoint will be quality of life (EQ-5L-5D). The intervention will last 12 weeks with a daily intake of omega-3 or placebo and a daily supervised rehabilitation programme using telerehabilitation.

**Discussion:**

This study suggests that SPMs supplementation combined with telerehabilitation may improve inflammation and symptoms like fatigue in Post COVID Condition patients. Both interventions have anti-inflammatory potential, and their combined use could enhance physical and mental health outcomes. This approach offers a promising strategy for managing Post COVID Condition symptoms.

**Trial registration:**

ClinicalTrials.gov NCT06063031

## Introduction

The World Health Organization (WHO) defines Post Covid Condition (PCC) as a syndrome characterized by “a history of probable or confirmed SARS-CoV-2 infection, usually three months from the onset of COVID-19, with symptoms that persist for at least two months and cannot be explained by an alternative diagnosis [1]”. It is estimated that at least 10% of the SARS-CoV-2 infected population develop PCC [2] also known as Long-Covid [3]. PCC shares similarities with other post-viral fatigue syndromes (Epstein-Barr or Cytomegalovirus) [4], such as chronic fatigue syndrome/myalgic encephalomyelitis (CFS/ME) particularly in terms of immune system dysregulation and persistent inflammation [5]. PCC is characterized by a broad spectrum of symptoms including, dyspnea, “brain fog”, anxiety, depression, gastrointestinal changes, and a general deterioration in quality of life [6], with fatigue being one of the major impairments for patients [7]. These symptoms suggest a possible overlap in the underlying pathophysiological mechanisms, particularly involving immune system dysfunction and chronic inflammation. However, the etiopathogenesis of PCC is probably multifactorial. Numerous studies suggest that viral RNA may in multiple organs or reactivate latent viruses such as EBV, which could exacerbate symptoms [5,8]. A crucial element of PCC is the presence of chronic hyperinflammation. The SARS-CoV-2 virus can trigger an exaggerated immune response, characterized by the release of proinflammatory cytokines including IL-6, IL-1, TNFα and reactive oxygen species. Some studies have attempted to detect the presence of T cells and NK lymphocytes in these patients with alterations in TCD4+ cells and TCD8+ cells [9,10].

Considering the inflammatory process, nutritional interventions -particularly those involving resolvins such as those derived from omega-3 fatty acids (EPA/DHA)-, are of interest. Omega-3 fatty acids serve as precursors of specialized pro-resolving mediators (SPMs), which play a critical role in resolving inflammation and restoring immune homeostasis by counteracting pro-inflammatory signaling pathways [11,12]. The specialized nutritional supplement used in this study is derived from fish oil containing standardized levels of SPMs derived from fish oil, that could be useful in PCC [13]. By supplementing SPM-rich formulations, it may be possible to modulate the inflammatory response and improve clinical outcomes in PCC patients [14]. In addition to nutritional intervention, the treatment of these patients should be multimodal, with pulmonary rehabilitation, physical rehabilitation and attention to psychological variables [15]. In fact, the WHO highlights the critical importance of rehabilitation for individuals with PCC, with physiotherapy playing a central role in patient recovery. A cohort study by Vanichkachorn et al [16,17], reported the efficacy of personalized rehabilitation programs in improving the overall condition of PCC patients, drawing parallels with treatment strategies used for CFS/ME. Other studies have also demonstrated the efficacy of tele-rehabilitation physiotherapy programs in enhancing post-COVID and Long COVID patients outcomes [18].

Therefore, the aim of this randomized controlled study will be to analyze the impact of a 12-week nutritional intervention with SPMs, combined with a telerehabilitation program, on the quality of life, pro-inflammatory balance, fatigue, dyspnea, respiratory strength, functional status, and psychosocial aspects in PCC patients, compared with a placebo supplementation with the same telerehabilitation program.

## Materials and methods

### Study design

**Fig 1:**
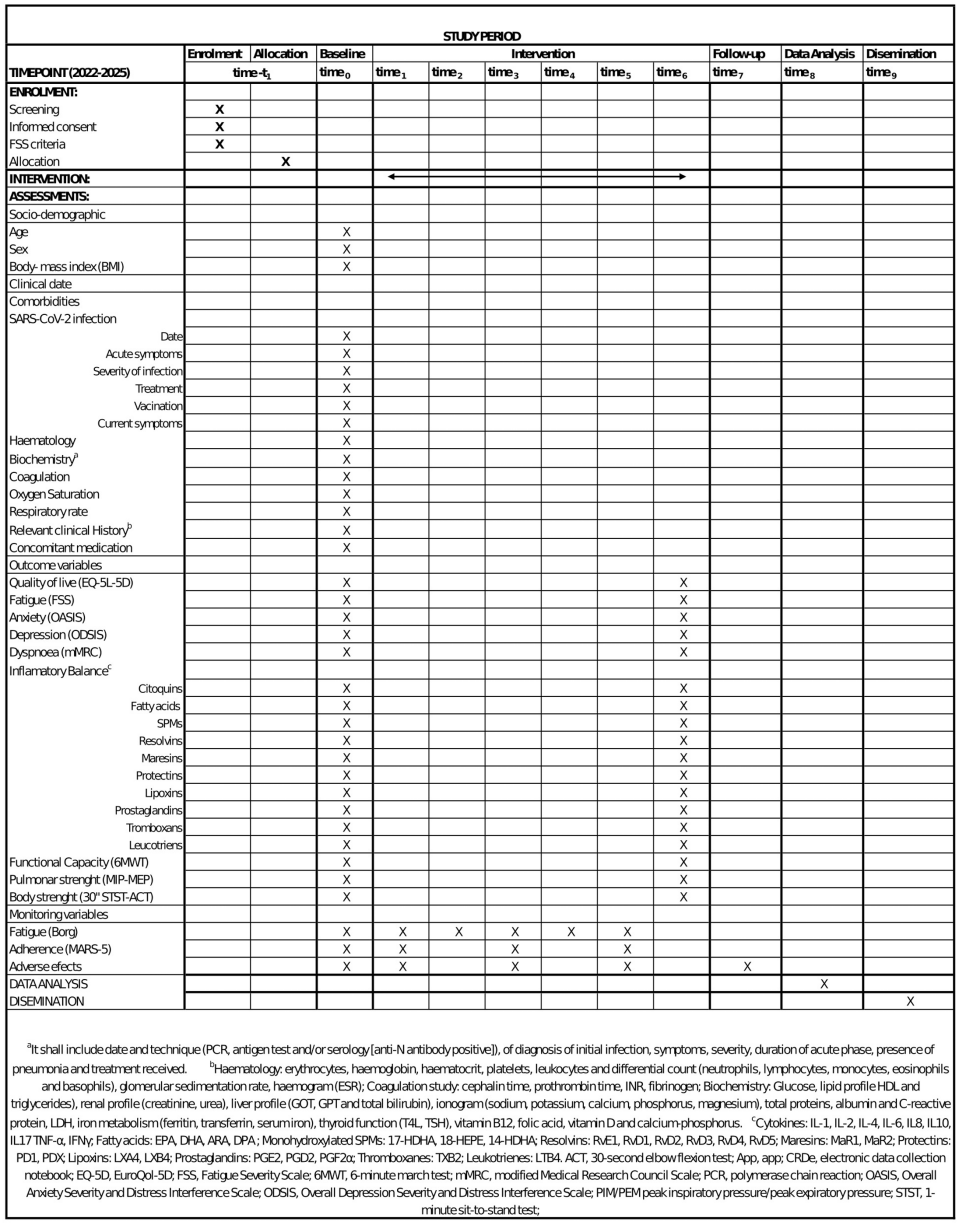
SPIRIT Schedule Enrollment.

ARACOV-2 will be a double-blind randomized control trial. This protocol has been designed according to the SPIRIT recommendations, approved by the Ethics Committee of Aragón (reference number: PI22/335), and registered at clinialtrials.gov (NCT06063031). Two parallel interventions will be compared: an experimental group (Nutritional supplement of SPMs and telerehabilitation) (NG) and a control group (placebo supplement and telerehabilitation) (PG). The duration of the study will be 12 months, with a 12-week intervention. Participants in the ARACOV project are actively engaged in the study, with enrollment anticipated to conclude by the end of 2024. Data collection is projected to be achieved by April 2025, paving the way for the obtained of study results in the latter half of 2025 (Fig 1).

### Setting and population

ARACOV-2 is part of a broader multicenter study known as “ARACOV” [19], a cohort designed to characterize patients with Post COVID Conditions (PCC), with people from three health centers of Aragón (Spain). Participants enrolled in ARACOV will be informed about the ARACOV-2 study and will provide with a participant information sheet (PIS) and study contact number. Recruitment will take place by the same research team in three Spanish health centers.

### Eligibility criteria

All potential participants will be informed before and will have to give their written consent to participate in the study. The inclusion criteria will be: 1) Aged 18–70 years; 2) Diagnostic criteria for having had COVID-19; 3) PCC symptoms for more than 12 weeks, from the end of the acute phase; 4) Fatigue >4 points on the Fatigue Severity Scale (FSS); 5) Independent locomotion. The exclusion criteria will be: 1) Neurological disease preventing them from following the program; 2) Respiratory failure: SaO2 < 90% or respiratory rate ≧30; 3) Rheumatic diseases or acute musculoskeletal injuries that contraindicates exercise; 4) Not daily access to the Internet; 5) Unable to follow oral and written instructions in Spanish; 6) Allergies to fish, shellfish, crustaceans or any of the components of the supplement; 7) Patients who have participated in or completed another clinical trial for the treatment of symptoms derived from COVID-19 in the last 4 weeks; 8) To take immunosuppressive drugs, corticosteroids or non-steroidal anti-inflammatory drugs in the last 2 weeks and 9) Being pregnant. The dropout criteria will be: 1) Pregnancy; 2) At the discretion of the researcher for safety reasons, and/or an adverse event; 3) Non-compliance or lack of adherence to the protocol for more than 2 consecutive weeks.

### Allocation and blinding

The randomization list will be conducted by the Contract Research Organization (CRO) in a stratified randomization stratified by age and sex using the EPIDAT4 program, a validated system that automates the randomization of treatment arms at the specified ratio, in this case in a 1:1 ratio (NG) or (PG). After baseline the data collection logbook (DCL) will assign a code according to these randomization criteria, which will correspond to the number of the supplementation batch to receive: Nutritional supplement or Placebo.

The treatment will be administered in a double-blind procedure. The allocation list will be kept by specific non-blind personnel from the CRO and the Promoter’s Quality Assurance Department, following good clinical practices. The assessors, the physiotherapist (intervention manager) and other investigators involved in the intervention will be blinded.

### Procedure

Patients who meet the criteria will be scheduled at their participating center. After signing the informed consent (IC) they will carry out the initial assessment.

At the baseline assessment, the nursing team will conduct a series of evaluations. Blood tests will be performed on all participants, and their oxygen saturation and respiratory rate will be measured. Participants will then complete six scales in a single room, a process expected to take approximately 30 minutes. Following this, they will proceed to the physiotherapy assessment, which will begin with the measurement of Maximum Inspiratory and Expiratory Pressure (MIP/MEP). Next, strength tests will be conducted, including the 30-second Arm Curl Test (30“ACT) and the 30-second Sit-to-Stand Test (30”STST). After a 10-minute rest period participants will undergo the 6-Minute Walk Test (6MWT) to assess aerobic capacity. This test will be conducted according to SEPAR guidelines in a 30-meter corridor [20].

In the final phase of the assessment, a physiotherapist will explain the prescribed exercise regimen tailored to each participant’s level and will also install the telerehabilitation mobile application (HEFORA) on the participant’s device.

**Fig 2:**
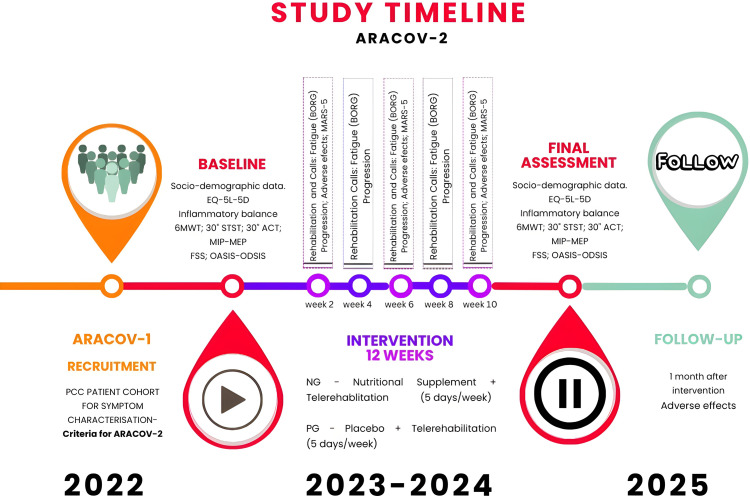
Study Design.

After randomization by the computer system, the nursing team will provide each participant with a supplement (Nutritional supplement or Placebo). The entire evaluation process will be repeated at the last visit, scheduled for day 84 ± 4, following the same procedures (Fig 2).

#### Procedures *during the* treatment period.

At weeks 2, 6, and 10 (±3 days), patients will be contacted by telephone to assess their adherence to the dietary supplement using the MARS-5 test and to monitor safety (Appendix 2 in S2 File). All symptoms reported by patients will be further assessed by a specialist in internal medicine and shall assess the intensity of an AA/unwanted effect and record it in the CCL, based on three intensity and relationship levels: mild, moderate and severe/ probable, possible and unrelated. All detected effects shall be reported to the health personnel for the correct follow-up of the patient. If any patients are identified as non-adherent during these intermediate check-ins, the significance of maintaining adherence will be emphasized to maximize health outcomes. Additionally, patients will receive biweekly calls (at weeks 2, 4, 6, 8, and 10 ± 3 days) to monitor their telerehabilitation treatment, during which the intensity and level of exercises will be reviewed and adjusted as necessary (Fig 2).

### Intervention

The treatment will take place for 12 weeks and will include both nutritional supplement and telerehabilitation. Participants must complete a therapeutic exercise programme five days a week via HEFORA telerehabilitation platform, which can be accessed at www.hefora.net or through the mobile app (HEFORA, Fisio Consultores, Zaragoza, Spain). The rehabilitation program will be designed following the recommendations of the NICE guideline [21,22] and the European and American Thoracic Societies [22]. It will include aerobics, strength and pulmonary rehabilitation, structured into three levels of difficulty, each featuring different exercises, except for [23] the pulmonary exercises (Appendix 1 in S1 File). Participants will be categorized into levels based on their fatigue measured with the FSS scores: those scoring 6.00 or higher will be in the first level, those with scores between 5.00 and 5.99 in the second, and those scoring between 4.00 and 4.99 in the third. Each participant will begin with 10 minutes of the prescribed aerobic exercises and three sets of strength exercises, performing as many repetitions as they can manage. The pulmonary exercises will remain consistent throughout the intervention, consisting of 2 sets of 10 repetitions each, with a target fatigue level of less than 3 on the Borg Scale.

For the first two weeks, all participants will perform the exercises with a fatigue intensity ranging between 3.00 and 4.00. After two weeks, this intensity will be increased to a range of 5–7. Progression in the exercise program will be assessed every two weeks during control calls. If any symptoms worsen, the participant’s progression in the program will be paused until the next assessment.

### Nutritional Intervention

Specialized Pro Resolving Mediators (SPMs) (17-HAD, 14-HAD and 18-HEPE) are a natural and standardized nutritional supplement selected to evaluate its efficacy in improving quality of life and other variables in patients with PCC after exposure after 12 weeks. The NG will receive the Nutritional supplement (500mg) enriched in SPMs, administered as 2 capsules after breakfast and 2 capsules after dinner. The Nutritional supplement with SPMs is a commercially available product. It contains an active fraction from fish oil, with standardized amounts of SPMs.

The PG will receive a placebo supplement composed of refined olive oil, gelatin and water which looks exactly like NG. The posology will be equal to the NG.

### Outcome measures

#### Primary outcome: Quality of life: EQ-5D-5L.

The EQ-5D-5L is a generic instrument for measuring health-related quality of life that has been validated [23] in Spanish and in PCC patients. It consists of two components: the levels of severity by dimensions and a visual analogue scale (VAS) on general health. The descriptive system contains five dimensions of health (mobility, self-care, activities of daily living, pain/discomfort, and anxiety/depression) and each of them has five levels of severity (From 1=No problem to 5=unable to perform). Participants indicate their perceived overall health on a VAS from 0 (“worst health I can imagine”) to 100 (“best health I can imagine”). The values obtained will be compared with the reference values [24]. Quality of life will be analyzed as a continuous variable by comparing the relevant statistics according to their distribution at the two points in time and as a qualitative variable by comparing the percentage of patients who improve their scores.

#### Secondary outcomes.

**Inflammatory and pro-resolving profile:** Behind the symptoms of PCC lies an unresolved inflammatory cascade. Since SPMs are considered to be pro-resolving inflammation, it was considered to analyze the change in this inflammatory profile [25–27]. The analyzed parameters will be: cytokines: IL-1, IL-2, IL-4, IL-6, IL-8, IL-10, IL-17A TNF-α, and interferon-gamma (IFN-γ); free fatty acids: EPA, DHA, arachidonic acid (ARA), (DPA) and monohydroxylated lipid mediators (LMs): 17-HDHA, 18-HEPE, 14-HDHA; resolvins (Rv): EPA-derived RvE1, RvE2 and DHA-derived resolvins (RvD): RvD1, RvD2, RvD3, RvD4, and RvD5; maresins (MaR): MaR1, and MaR2; protectins: PD1 and PDX; lipoxins (LX): LXA4, and LXB4; prostaglandins: PGE2, PGD2 and PGF2α; thromboxane TXB2; and leukotriene LTB4.

Blood samples for cytokine analysis, will be centrifuged for 15 minutes at 1000 x g. Then, the serum will be removed, aliquoted and stored at −80°C until cytokine measurement. IL-6 will be measured by enzyme-linked immunosorbent assay using the Quantikine® HS Human Immunoassay Kit (HS600C, R&D systems) according to the manufacturer’s recommendations by using a Biotek Synergy HT Microplate Reader. The rest of the cytokines will be determined by using the Human XL Cytokine Luminex® Kit Performance Assay (FCSTM18B, R&D Systems) according to the manufacturer’s recommendations and analyzed with a LABScan 100 Multiplexing Analyzer System (Luminex Corporation).

Plasma and serum samples for free fatty acids and lipid mediator determinations will be collected and processed as previously described by Regidor et al. 2022 [28]. In brief, peripheral blood samples will be collected and immediately centrifuged at 120 g for 20 min with free stop. Plasma supernatant will be transferred to 1.5 mL tubes, topped with nitrogen gas, capped, and immediately stored at −80°C for further processing. Serum samples will be generated as Norris et al. 2017 [29]. In brief, after an overnight incubation at 37°C, the same protocol described for plasma will be applied. Then, for LMs extraction, a solid-phase extraction (SPE) method will be performed as follows: 4 mL of methanol containing 500 pg. of each internal labelled standard (d8-5-HETE, d5-RvD2, d5-LXA4, d4-LTB4, and d4-PGE2, Cayman Chemical Company) will be added to enable ulterior samples LMs quantification. Calibration curves will register using synthetic and authentic LM mixtures. After that, a protein precipitation phase will take place (−80°C, 30 min) and posterior centrifugation at 2000 g, 10 min, 4°C. In addition, the supernatant will be acidified to pH = 3.5 just before loading onto a conditioned-SPE column (Biotage) and pH neutralized with MilliQ water, followed by an n-hexane wash step. After that, lipid elution will take place with 9 mL of methyl formate. Finally, extracts from the SPE will be dried under a stream of nitrogen and resuspended again in 50μL methanol/water (1:1) before injection into the LC-MS/MS system. The full method and acquisition LC-MS/MS parameters were previously published [30].

The remaining secondary variables were selected based on scientific literature [18,31–33]

#### Fatigue.

Fatigue will be measured with the FSS [34], a self-reported scale that allows assessing the severity of fatigue as a sense of physical tiredness, muscle weakness and lack of energy. It has been validated in post-COVID patients [35]. It is composed of 9 items with scores ranging from 1= strongly disagree to 7= strongly agree. The higher the number, the greater the severity of fatigue. The most common cut-off point is a mean score of 4 points, considering equal or more than 4 as severe fatigue [36].

#### Dyspnea.

The dyspnea will be measured with the modified Medical Research Council (mMRC) [37]. This scale has already been used in other studies to assess the degree of dyspnea in post-COVID-19 patients [38]. This scale allows determining the magnitude of the dyspnea that the patient presents by scoring from 0 = absence of dyspnea to 4 = does not leave the house due to dyspnea.

#### Respiratory strength.

Measurements of maximum inspiratory pressure (MIP) and maximum expiratory pressure (MEP) can help evaluate respiratory muscle weakness in PCC patients [39]. This test is widely used to non-invasively evaluate respiratory muscle strength in clinical practice [40] and presents the cut-off point to detect muscle weakness for the Spanish population [41]. MIP/MEP will be measured based on the ATS/ERS 2002 guidance using the MicroRPM CareFusion pressure meter with individual antibacterial filters.

#### Functional status.

- 6MWT is a sub-maximal exercise test used and recommended to assess the maximum distance possible for six minutes, in a 30-meter corridor, allowing the patient to rest as needed. It has been shown to be reliable [42–44]. The distance covered by each participant will be compared with the estimated distance for their gender, weight, and age according to the Troosters equation [45].

- 30“STST is part of the Senior Fitness Test (SFT) designed by Rikli and Jones [46–48] and it will be used as a stand-alone test, especially to assess weakness in respiratory patients who have passed COVID-19 [49] and it has been shown to be reliable in adults with respiratory pathologies [50–53]. For the 30”STST, participants will sit in a chair with their feet flat on the floor and arms crossed over their shoulders, performing as many squats as possible within 30 seconds [47].

- 30“ACT is part of the SFT and is also used as a stand-alone test to assess strength. It has been shown to be reliable [51,52] in deconditioned patients and in the elderly population and used in Long COVID patients [31]. The higher number of repetitions the better strength [47,54]. During the 30”ACT, picipants will sit in a chair and perform as many elbow flexions as possible within 30 seconds using their dominant arm, with women lifting a 2 kg weight and men a 4 kg weight.

#### Psychosocial factors.

Psychosocial factors will be assessed by using Overall Anxiety and Depression Severity and Impairment Scales, OASIS and ODSIS, respectively. These scales have been used in studies with COVID-19 patients and have been validated in Spanish [55,56]. Both are two self-report questionnaires consisting of 5 and 9 items graduated in a Likert scale from 0= never to 4= all time, that assess the frequency, intensity, interference and severity of anxiety and depression on the activities of daily life. The total score range goes from 0 to 20, the highest score the highest involvement of anxiety or depression in patients. The cutoff scores will be the following: 15 points for OASIS and 12 for ODSIS.

#### Other outcomes.

**Clinics:** Comorbidities, COVID-19 diagnosis, initial symptoms, severity of infection, diagnosis, current treatment, vaccination, and blood data (hematology, coagulation, biochemistry) have already been registered in the ARACOV study (which was mentioned above).

#### Adherence.

Adherence to the telerehabilitation program will be recorded through the mobile application by pressing the “done” button for each exercise in the application itself. It will be coded as follows: 0= if the activity was not done, 1= if they have done some exercises that day, 2 = if they have all exercises that day. Those who complete more than 80% of the sessions (code 1 or 2) will be considered very adherent. “Non-adherent” will be understood as those who completed less than 20% of the sessions [57]. The rest will be considered “partially adherent.”

To evaluate adherence to the supplement, the patient will return the leftover product at last visit and the % adherence will be calculated according to the following formula: % Adherence = (Units taken/ Theoretical units to be taken in x days)*100. Patients will be considered adherent when the percentage adhesion is between 90–100%.

#### Sample size calculation.

**Fig 3:**
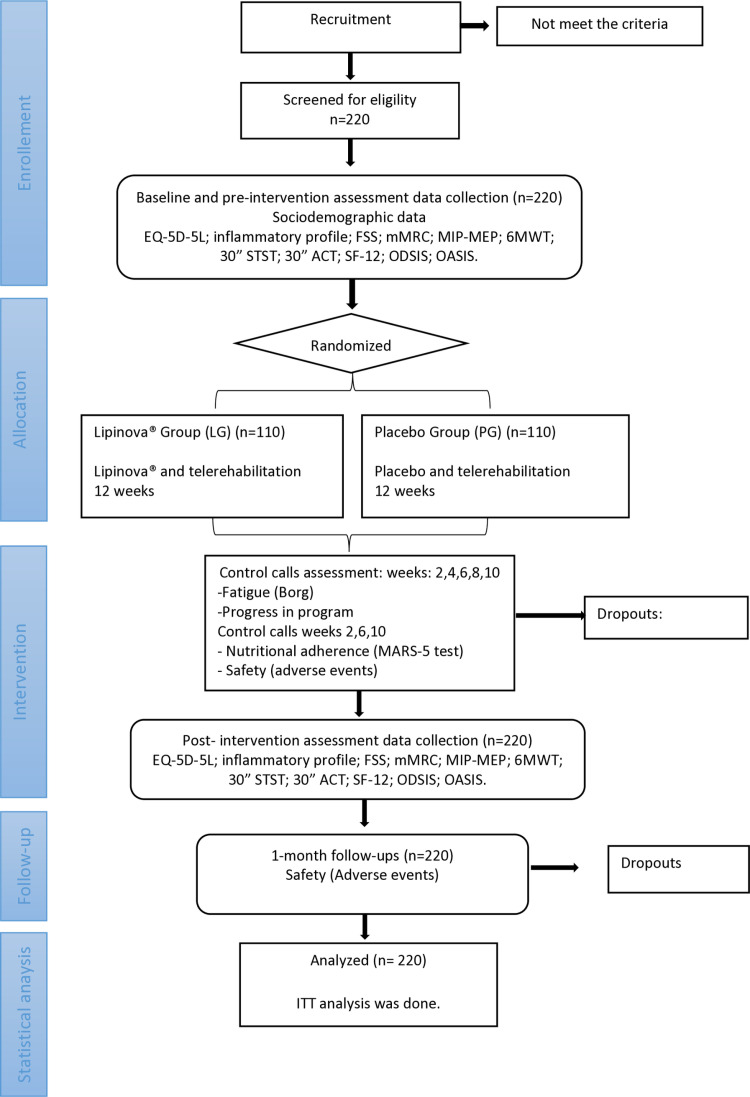
Flow Chart.

To estimate the sample size, a hypothesis test comparing the proportions of independent groups (PG vs. NG) assuming an alpha risk of 0.05 and a beta risk of 0.2 in a bilateral contrast requires 110 subjects in the placebo group and 110 subjects in the Nutritional supplement group to detect the difference in percentage of patients that will improve their EQ-5D-5L after intervention between the two groups, expected to be 30% and 50%, respectively, as statistically significant, and including a 15% loss estimated. Likewise, to assess the improvement in the pro inflammatory balance considering in this case the proportion of patients with change between pre-post intervention balances in each of the groups the estimated sample size will allow a difference of 20% or more between the two to be declared statistically significant, with a confidence level of 95% and a power of 80%. The estimation was carried out using the GRANMO sample size calculator, version 7.12 (Fig 3).

#### Statistical analysis.

Statistical analysis will be performed with IBM-SPSS Statistics software version 25 or later. Quantitative variables will be described as mean and standard deviation (SD) if the variable follows a normal distribution or as median and interquartile range for non-normal data. For categorical data absolute and relative frequencies will be computed. The Kolmogorov-Smirnov test will be used to determine the normality of the data. Intra-group variables will be measured with the T-Student test for related samples or the paired samples Wilcoxon test for non-parametric alternative. For inter-group comparations non-parametric analysis will be performed when a non-normal distribution is assumed, using the U Mann Whiney test for comparisons between groups and T-Student test for normal data. Dichotomous variables will be analyzed using the chi-square test. A significant level of 95% will be assumed (p <0.05). If there are more than 15% dropouts, an intention-to treat (ITT) analysis will be performed. Effect size will be calculated using Cohen’s d to determine clinical significance: negligible, small, medium and large differences will be reflected in effect sizes of < 0.2, 0.2–0.5, 0.6–0.8 and > 0.8, respectively.

## Discussion

Based on our literature review, this is the first study that will combine a dietary supplement based on SPMs with telerehabilitation to reduce the symptoms of patients with PCC. The effectiveness of a nutritional over only a physiotherapeutic intervention would open a field of intervention for these patients who still do not have an effective treatment.

Statistical improvements in quality of life are expected in the Nutritional supplement group to be linked to the improvement of the inflammatory profile although changes are expected in both groups. Specialized nutrition with SPMs might have favorable effects on PCC symptoms due to the potential mechanisms of action for omega-3 acids and their metabolites [25,58]. These actions will result in clinical improvement and therefore in the quality of life perceived by patients. In this regard, Pavlidou’s review confirms that this type of supplementation is beneficial before and during COVID-19 disease and encourages studies like this one to show its effect in patients with PCC [59]. In addition to the nutritional supplement, pulmonary rehabilitation and controlled aerobic and strength training seem to be the most effective interventions used to improve the symptoms of PCC [60]. Furthermore, telerehabilitation has already been shown to be effective in improving quality of life in a similar way to face-to-face treatments, as reported by Dias et al. 2021 in a systematic review [61].

It is expected to have a reduction of long-lasting inflammation in NG for the anti-inflammatory effect of omega-3 [62] by blocking the inflammasome activation and pro-inflammatory cytokine production, thereby restoring tissue homeostasis; improving innate immunity; and reducing oxidative stress by controlling the activity of related enzymes and the generation of free radicals (ROS) [63]. Although the effectiveness of SPMs in reducing inflammation in these patients is not yet proven, in vitro studies show that both Resolvin D1 and Resolvin D2 are potent in reducing the production of pro-inflammatory cytokines, showing the potential role of lipid mediators in the treatment of PCC [64]. The favorable effects of physical exercise on the reduction of inflammation, as well as the modulation of cytokines, have been widely reported in different fields [65,66]. Therefore, improvements can be expected in both groups, although NG will show greater changes, due to the sum of two interventions with anti-inflammatory potential.

This expected improvement in the inflammatory balance in NG will be linked to major improvements in fatigue and dyspnea levels, functional status and psychosocial variables. The presence of sustained low-grade inflammation in these patients is a limitation that physical rehabilitation faces today in improving its effectiveness, especially as this inflammatory cascade leads to an increase in perceived fatigue [67]. It is already known that the fatigue characteristic of PCC is similar to chronic fatigue syndrome, which is also characterized by emotional and mental disturbances. Fatigue severity has been significantly correlated with increased monocytes, up-regulation of CCL2, CCL7 and SERPINB2 gene levels in monocytes, increased serum galectin-9 and increased CD8+ T-lymphocyte counts. Likewise [68], other nutritional supplements such as L-arginine plus vitamin C or anhydrous enol-oxaloacetate have been shown to be effective in controlling/relieving PCC and thus, a reduction in fatigue [69–71]. Other studies have established the benefits of vitamin and mineral supplements, particularly vitamin C, vitamin D, zinc and selenium, or multivitamins with flavonoids such as quercetin and bromelain [67].

In terms of respiratory strength and functional status, positive changes can be expected in both groups, even if they will be particularly relevant for NG. There are no studies on the benefits of omega-3 supplementation for PCC, but a recent meta-analysis has shown that there are benefits for muscle mass and performance (walking speed) in older people [72]. As for other supplements, a small group of COVID-19 survivors in a pre-post study showed an improvement in muscle strength and physical performance with a dietary supplement containing 19 nutrients, including amino acids such as arginine and carnitine, minerals, B-group vitamins, and plant extracts. Although the study showed positive results, there was no control group, and no exercise program like ours was proposed [73]. In addition, according to the latest systematic review, pulmonary rehabilitation has proved to be effective in the different stages of COVID-19, which leads us to assume that MIP-MEP, dyspnea, and functional status could improve in both groups [74].

Related to the inflammatory mechanism and mental health, kynurenine, a metabolite increased in COVID-19 patients, is associated with depression states and contributes to oxidative damage in the brain (neuroinflammation). In contrast, kynurenic acid cannot cross the blood-brain barrier, protecting the brain from depression [75,76] Reducing inflammation with EPA/DHA fatty acids combined with exercise can increase the conversion of kynurenine into kynurenic acid in the skeletal muscle and improve emotional symptoms. Although a recent review has concluded that omega-3 fatty acids deficiency is associated with an increased risk of mental disorders [77], there are currently contradictory results on the effectiveness of omega-3 on depressive symptoms, with variable factors such as dosage and patients’ baseline diet being the cause of these contradictions [78]. However, we expect that the mental health of patients with PCC of NG will improve significantly thanks to nutritional supplementation and telerehabilitation, as exercise has been proposed as a good strategy for neurological recovery from COVID-19 sequelae.

For all the above reasons, the combination of SPMs supplementation and telerehabilitation would be a promising strategy to improve the physical and mental health of patients with PCC.

### Strengths and limitations

The limitations of this study may be the problems of patient recruitment, as the sampling is of convenience, there may be a bias of participation of those patients who are more symptomatic and better adherent due to their interest in participating, and by design, we cannot know the effect of tele-rehabilitation versus only giving recommendations or the effect of nutritional supplement enriched in SPMs in isolation. Furthermore, because of the multiple contrasts due to the large variety of variables, a significance of 95% could increase the type I error.

## Supporting information

S1 File Appendix I. Rehabilitation Program by HEFORA.
(PDF)

S2 File Appendix II. Adverse Events Reporting.
(PDF)
