# Supplementary material for: ARACOV-02. Specialized nutritional intervention and telerehabilitation in patients with long COVID: Protocol of a randomized controlled trial
Source: PLoS One. 2025 Apr 29;20(4):e0321811. doi: 10.1371/journal.pone.0321811 (PMC12040102; doi:10.1371/journal.pone.0321811)
Supplement: S1 File — Rehabilitation Program by HEFORA. (PDF) [file pone.0321811.s001.pdf]

**Appendix I. Rehabilitation Program by HEFORA**

1.- RESPIRATORY (daily) 2 sets x 10 repetitions Fatigue less than 3/10 Borg

1.1.- Post-inspiratory apnoea 5", followed by exhalation with pursed lips

1.2.- Breathing with arm movements to expand the rib cage

1.3.- Blowing through a straw into a bottle of water

2.- STRENGTH (3 days a week with at least one rest day) 3 sets of 8/ 3 x 12/ 3x 15 at each level Fatigue level: - 3-4 Level 1 - 5-6 Level 2 - 6-7 Level 3

2.1. a. Standing, with hands resting on a chair or stable table, stand on tiptoe with both legs in a slow and controlled manner

2.1.b. Sit down and stand up from a chair with hands crossed over shoulders

2.1.c. Gluteal bridge on the floor

2.1.d. Wall push-ups on a slightly inclined plane

2.1.e. Rowing with a rubber band in a doorway (scapular retraction+shoulder extension+elbow extension)

2.1.f. Wall incline plank lifting alternate knees 30"

2.2.a. With two bottles of water in your hands, standing, bend your knees slightly, extend them and stand on your toes with both legs in a slow and controlled manner

2.2.b. Shallow squats with hands resting on stable equipment or back against the wall

2.2.c. Gluteal bridge with alternate leg lifts 2.2.d

2.2.d. Wall push-ups 45° incline with hand to opposite shoulder after extension

2.2.e. Incline barbell rowing

2.2.f. Incline plank on a stable piece of furniture lifting knees 30"

2.3.a. With something heavy in both hands, pick it up off the floor and lift it over your head with your arms while standing on your tiptoes

2.3.b. Deep squats accompanied by arm movements

2.3.c. Hip thrust on couch, bed ...

2.3.d. Burpee low tempo without final jump

2.3.e. Posterior push-ups on a stable piece of furniture 2.3.f. Inclined plank on a stable piece of furniture lifting knees to touch the opposite hand 30"

3.- AEROBIC (5 days a week) 10 to 30 minutes at a time with a progressive increase in speed. Fatigue level: - 3-4 Level 1 - 5-6 Level 2 - 6-7 Level 3

3.1.a. Walking

3.1.b. Walking by adding hills or stairs

3.1.c. Fast walking

3.2.a. Walking up a few flights of stairs

3.2.b. Swimming at the marked intensity

3.3.a. Jogging

3.3.b. Static jumping

3.3.c. Any other sport at the marked intensity

3.3.d. Any other sport at the marked intensity
