# Supplementary material for: ARACOV-02. Specialized nutritional intervention and telerehabilitation in patients with long COVID: Protocol of a randomized controlled trial
Source: PLoS One. 2025 Apr 29;20(4):e0321811. doi: 10.1371/journal.pone.0321811 (PMC12040102; doi:10.1371/journal.pone.0321811)
Supplement: S2 File — Adverse Events Reporting. (PDF) [file pone.0321811.s002.pdf]

Appendix II. Serious adverse events reporting/ serious unintended effects / serious unintended effects by the investigator to the promoter

In order to meet the requirements of the regulatory authorities, adverse events classified as serious adverse events (SAEs) and serious undesirable effects require immediate reporting to the Sponsor.

Any Serious Adverse Adverse Event or Serious Undesirable Event, related or unrelated to the investigational supplement, should be reported by the health care personnel to Dynamic (Evidenze Clinical Research), completing all the information in the corresponding section through the CRDe. At the same time, the investigator must complete the specific form for reporting AAG or Serious Undesirable Effect and send it by email to farmacovigilancia@dynasolutions.com. Upon receipt, Dynamic (Evidenze Clinical Research) will ensure that there is no missing information to be included in the form and will send it once its review has been verified to Solutex at the following email address: ghiguera@solutexcorp.com and mhawkins@solutexcorp.com.

In any case, the communication of any AAG or serious undesired effect must be made within 24 hours of the investigator becoming aware of it and within 24 hours of Dynamic (Evidenze Clinical Research), to Solutex, with a record of its review.

In the event that the investigator becomes aware of a serious AGE or Serious Undesirable Effect (regardless of its relationship to the investigational supplement) occurring within 2 days after discontinuation of the investigational product, the AGE should be reported in accordance with the procedures specified in this protocol. In case of death, if an autopsy is performed and the autopsy report is available, an anonymised copy should be provided to Solutex.

CONTACTS FOR NOTIFICATION OF AAG IN SPA

Monitoring: Monitor			DYNAMIC SCIENCE S.L.U (Evidenze Clinical Research),		
**Address**			C/ Caléndula, 93 Edificio K Minipark III		
**CP**	28109	**Population**	Alcobendas	**Provincia**	Madrid
**Telephone**	+34 914561105		**Fax**	+34 914561126	
**email**			farmacovigilancia@dynasolutions.com		
